# Intrusion of quantum crystallography into classical lands

**DOI:** 10.1107/S2052520625000782

**Published:** 2025-03-06

**Authors:** Sizhuo Yu, Jean-Michel Gillet

**Affiliations:** a Université Paris-Saclay, CentraleSupélec, CNRS Laboratoire SPMS F 91190Gif-sur-Yvette France; Warsaw University, Poland

**Keywords:** quantum physics, quantum crystallography, Wigner function, reduced density matrix, Compton scattering, high-resolution X-ray diffraction, phase space, momentum space

## Abstract

This article, written on the occasion of the International Year of Quantum Science and Technology, explores the development of alternative approaches to pure-state *N*-electron wavefunctions in quantum physics. It focuses on Quantum Crystallography tools and explains how the combination of X-ray coherent-elastic and incoherent-inelastic scattering data has enabled the description of mean-electron’s quantum behaviour in phase space. The article gives a numerical example using a urea crystal to demonstrate the attainability of recovering a one-electron reduced density matrix and its associated reduced Wigner function. It emphasizes the importance of momentum-space measurements in obtaining a more accurate phase-space picture of electron quantum physics in crystals.

## Abstract 2

One hundred years after the quantum theory established position and momentum as incompatible quantities, quantum crystallography offers a way to visualize electron phase space behaviour in crystals.

## 1. Introduction

This paper contributes to the celebration of 2025 ▸ as the International Year of Quantum Science and Technology. It has indeed been one hundred years since the mathematical foundations of quantum physics were first proposed by Schrödinger, Heisenberg, Jordan and Born. We here adopt the biased perspective of Quantum Crystallography (QCr) to describe the reduced density matrix or the Wigner function as alternative approaches to the *N*-electron wavefunction and what progress has been made in their experimental quest. First, we explain how and why these quantities were defined and relate to electron density probability distributions in solids and molecules (Section 2). In the one-electron case, we then expose their connections to two common experimental techniques (Section 3). In Section 4, the main strategies for retrieving density matrices or Wigner functions from experimental measurements are revisited, distinguishing the pure-state problem from that involving a statistical mixture of states. Finally, in Section 5 we summarize our latest attempts to recover one-electron reduced density matrices and Wigner functions using a combination of data from the scattering techniques described in Section 4. We thus claim that it is possible to give a quantum representation of electron behaviour in the (once-believed) classical land of phase space.

## 2. Early alternatives to the wavefunction

A stay in the great outdoors is often beneficial for the body and the mind, whether to escape an allergy or the demands of daily life. We can imagine that the pure air of Heligoland was as favourable to W. Heisenberg (Cassidy, 1992 ▸) as that of the Swiss Alps was to E. Schrödinger (Moore, 1989 ▸). In the summer of 1925, inspiration gained by Heisenberg in the pollen-free air of the North Sea rapidly induced the ‘three-man paper’ with M. Born and P. Jordan. Thereby the mathematical basis to a new matrix formulation of quantum mechanics was established (Heisenberg, 1925 ▸; Born & Jordan, 1925 ▸; Born *et al.*, 1926 ▸). Schrödinger’s wave picture emerged from the snow the following winter, in the picturesque setting of Arosa (Schrödinger, 1926*a* ▸; Schrödinger, 1926*b* ▸). Rigorous proof of the mathematical equivalence of both formalisms only appeared in the next decade (von Neumann, 1932 ▸; Van Hove, 1958 ▸).

Interestingly, no sooner had Schrödinger found an evolution equation for his ‘Ψ-(wave)function’ than some of his contemporaries began to worry about the limits of this approach.

### 2.1. Density operator and density matrix

J. von Neumann (soon to be followed by P. Dirac and L. Landau) found that, if real systems are not isolated from their environment, one should consider a mixture of quantum states (von Neumann, 1927 ▸; Duncan, 2024 ▸; Dirac, 1929 ▸; Dirac, 1931 ▸) according to the laws of statistical mechanics. This description was easily achieved when a single wavefunction was replaced by a density operator. This operator is constructed from a convex combination of state projectors (using Dirac’s notation):
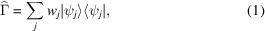
where 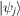 are quantum pure-state kets and 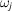 are their statistical weights in the mixture. When dealing with a closed system at a fixed temperature imposed by a thermostat, the weights are mere canonical probabilities if the 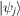 are the eigen-kets of the Hamiltonian. For any observable property represented by operator 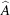, the mean value is thus computed from the trace:
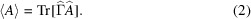
It was acknowledged early that two different types of mean values were thereby simultaneously computed: a ‘quantum’ expectation value, for each state, and a ‘classical’ (statistical) physics mean value, mixing all the quantum states. Using a single density operator over an infinite list of wavefunctions can thus be a convenient option when the system is expected to be in a non-pure state. If the choice is that of the density operator, time evolution is given by the von Neumann equation:
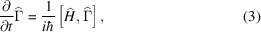
where 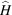 is the system’s Hamiltonian.

The non-commutation of position and momentum operators forced every quantum mechanical description to choose a side. The most common way of representing quantum states or density operators became that of position representation so that a wavefunction or a density matrix became the short name for the projection of the quantum state ket onto the position space, 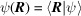, or the expression of the density operator expressed in the position operator’s eigenbasis 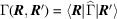, respectively.

### 2.2. A phase-space quantum picture

However, not everyone was ready to give up the convenience of phase space. Obtained from the direct product of position and momentum spaces, it had become the natural territory where classical states had progressively settled since R. Hamilton, J. Liouville, then L. Boltzmann and J. C. Maxwell (Nolte, 2010 ▸). In the early 1930s, when looking for an approximate method to include quantum effects in thermodynamics, E. Wigner (Wigner, 1932 ▸) suggested that the following function could bear all the required properties to combine position, 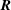, and momentum, 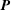, components:
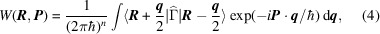
where *n* is the number of degrees of freedom of the system, generally 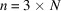, and *N* is the number of particles in the system. Therefore, 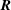 and 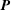, respectively, represent the position and momentum vectors in an *n*-dimensional space.

From its definition, it can easily be seen that the Wigner function, 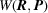, is just as adapted to the description of systems in a mixture of quantum states as the density operator 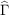 or the density matrix 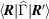. However, it has been shown that the Wigner-function picture is also equivalent to the Schrödinger description (Tatarski, 1983 ▸), since it also obeys an evolution equation:
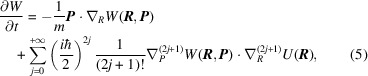
where 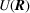 is the potential energy term of the Hamiltonian. Unlike the wavefunction and the density matrix, 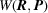 is a real-valued function. The Wigner-function approach was rapidly adopted when the purpose was to study the limits between classical and quantum physics. It is immediately obvious that if only the first term of the expansion is retained in (5), the Planck constant vanishes from the evolution equation and the classical Liouville equation is recovered. Going further, it would thus be tempting to interpret the Wigner function as a phase-space probability distribution since the marginal integrals yield the electron probability density distributions in position and momentum spaces, respectively:
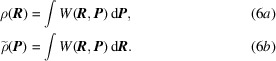
However, using definitions (4), it is clearly seen that for two orthogonal pure states, *i.e.*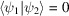, the product of their Wigner functions 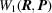 and 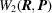 obeys the following relation:
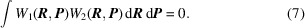
This implies that the Wigner function should be expected to take negative values in regions of phase space. This last property disqualifies the Wigner function from being a proper density probability function in phase space, despite relations (6). Apart from this ‘negativity *faux pas*’, 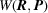 can be employed to compute mean values for any quantum observable 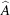:
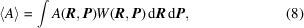
provided that the phase-space expression of 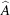 is defined using the Weyl transform (Weyl & Robertson, 1950 ▸):
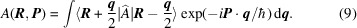
Consequently, there is no reason to abandon phase space as a possible playground for quantum physics.

### 2.3. Reduction of the problem to a mean pair or a mean single electron

Like for any pure-state wavefunction, the density matrix or Wigner function description of an assembly of *N* electrons can rapidly become too complex. As stated by Coulson, ‘a conventional many-electron wavefunction tells us more than we need to know’ (Coulson, 1960 ▸). Indeed, no known physical quantity involves more than two electrons at a time. Therefore, using (2), computing the quantum expectation value of such an observable from the density operator amounts to evaluating
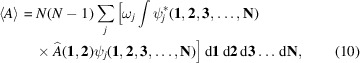
where the bold numbered variables include the spin and the position (or the spin and the momentum) for each particle. This expression can be rewritten as a two-step process:
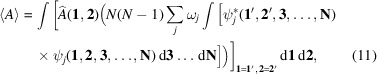
where operator 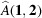 first acts on unprimed variables, before primed and unprimed variables are set equal. Formulation (11) demonstrates that there is no need for an all-electron density matrix, and a partial trace over the 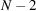 electrons is just as useful (Husimi, 1940 ▸; Haar, 1961 ▸). Defining the two-electron reduced density matrix as
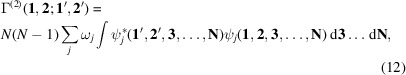
we obtain
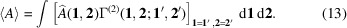
One can thus see that the reduction of the problem is equivalent to considering a mean electron pair, which carries all the quantum behaviour of the assembly.

In many cases, the problem can be simplified further by considering only the one-electron reduced density matrix (1-RDM):
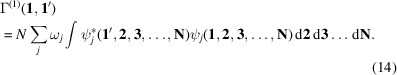
If the spin value is not relevant, a spin-traced 1-RDM becomes a mere function of the mean-electron’s position 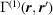 and the diagonal elements of the spin-traced 1-RDM yield the electron density distribution 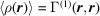, where 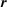 now refers to a single electron position (the mean electron).

Recourse to a 1-RDM description was already envisaged by Dirac in the very early times of quantum theory in the pure-state case when discussing the mean-field treatment of the atom (Dirac, 1930 ▸). A similar reduction of the problem can be carried out for the Wigner function approach. The one-electron Wigner function will be referred to as the 1-Wigner.

The main advantages of using the density matrix or, equivalently, the Wigner function formulation over the wavefunction are thus twofold. Firstly, since all observable operators are linear, statistical mixtures of quantum states are naturally accounted for in any mean-value computation. Secondly, the possibility of reducing the problem to a mean pair, or even a mean electron (reduced Wigner or reduced DM), without losing any accuracy in the quantum description, significantly simplifies the modelling and the graphical representation of the distributions.

## 3. Quantum behaviour from crystallography scattering techniques

The possibility of obtaining an accurate *N*-electron wavefunction from experimental X-ray data is still debated within the QCr community. Nevertheless, there is no doubt that the strong interplay between wavefunctions and experimental measurements has been a cornerstone of quantum physics since the early ages. From what precedes, it emerges that density matrices (at most 2-RDM) and their Wigner counterparts are the Holy Grail when it comes to predicting or describing the electronic properties of systems. Given the theoretical complexity and the sparsity of proper information, the quest for an experimentally derived 2-RDM or 2-Wigner has been regularly postponed. Consequently, we will only report on the progress achieved for the 1-RDM and 1-Wigner. The previous section demonstrated that the (reduced) Wigner function possesses exactly the same properties as the (reduced) density matrix and that the two descriptions are equivalent. Therefore, we will mostly refer to the 1-RDM case.

Expressions (2) and (8) and their one-electron versions clearly show that any experimental measurement should enrich our knowledge of the density matrix. For the 1-Wigner, the problem is a special class of quantum (crystallographic) tomography problem relying on Born’s rule, similar to that which has been practiced for photon states (as first proposed by Vogel & Risken, 1989 ▸) and expanded to a single electron (Jullien *et al.*, 2014 ▸). As it turns out, two very different scattering techniques, well established in the crystallography community, have long been known for containing detailed information on electron probability distributions. As a general remark, it is worth mentioning that these two specific techniques, belonging to the elastic and inelastic scattering regimes, provide symmetric approaches to both sides of phase space as they respectively focus on the position and momentum representations. The next section gives the essential aspects of these measurements and how they relate to the 1-RDM or the 1-Wigner.

### 3.1. A position representation of electrons by diffraction from single crystals

The diffraction of all kinds of particles was instrumental in the development of the quantum physics theory. Many are aware that the Laue–Friedrich–Knipping experiment killed two birds with one stone.

An X-ray interference pattern, resulting from scattering by crystalline ZnS (Friedrich *et al.*, 1913 ▸), was strong evidence that X-rays could exhibit wave-like behaviour and, concurrently, confirmed that crystals are indeed periodic arrangements of atoms. After the Braggs had demonstrated the usefulness of X-ray diffraction for determining a crystal’s atomic structure (Bragg, 1913 ▸), Debye was among the first to state that, with such a new technique, ‘the experimental investigation of the scattered radiation, especially with light atoms, seems […] of increased interest, as it should then be possible to experimentally determine the specific arrangement of electrons in the atom. Such an investigation therefore has the significance of an ultramicroscopy of the interior of the atom’ (Debye, 1915 ▸). Going further, Compton could not resist hoping for ‘more definite information concerning the distribution of the electrons in the atoms…" (Compton, 1915 ▸). At that time, Schrödinger was still on the war front and had not yet invented his ‘Ψ-function’.

Thus, even if ‘distribution’ could not yet be associated with ‘probability density’ [Born’s probabilistic interpretation only appeared in 1926 ▸ (Wheeler & Zurek, 1983 ▸)], in the same issue of *Nature*, W. H. Bragg replied that the density could be ‘analysed by Fourier’s series’. Once the basic formalism had been put forward by Heisenberg and Schrödinger in 1925, it was almost a natural consequence to read the 25-year-old L. Pauling stating in a letter, ‘I think it is very interesting that one can see the Ψ-function of Schrödinger’s wave mechanics by means of the X-ray study of crystals. This work should be continued experimentally; I believe that much information regarding the nature of the chemical bond will result from it’ (Pauling, 1926 ▸). Although it turns out now that Pauling was overly optimistic, there is no doubt that X-ray diffraction (XRD) was identified very early as an indispensable tool for studying the quantum behaviour of electrons in crystals. The electron distribution was obviously a major concern, but still restricted to the position component of phase space.

Formally speaking, the connection to the density matrix problem is straightforward. Whatever the projectiles employed, photons, neutrons or electrons, diffraction is an elastic coherent process. Therefore, if the target is periodic, every time the Bragg condition is fulfilled we can hope to have access to the Fourier transform of some function of the distribution density of the scatterers. Since electron behaviour is our main concern, one can consider X-ray photons as the most pertinent probes as they give access to the X-ray structure factors (XRSFs) 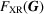, thereby to a partial representation of the 1-RDM, *i.e.* its diagonal elements 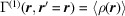 (14), or a projection of the 1-Wigner 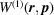 (6*a*[Disp-formula fd6]):
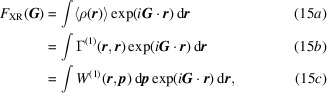
where 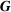 is the scattering vector.

When electron probes are used, the structure factors are the Fourier transform of the electrostatic potential (Spence, 1993 ▸; Ogata *et al.*, 2008 ▸). When polarized neutrons are employed, the key quantity is a flipping ratio (Gillon & Becker, 2012 ▸), which eventually yields the Fourier transform of the spin magnetism density distribution, created by the unpaired electrons 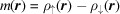.

Given its paramount impact on our understanding of the chemical bonding mechanism, reconstruction of the one-electron density distribution 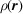 from a limited set of XRSFs has become a prominent task at the very heart of Quantum Crystallography (Genoni *et al.*, 2018 ▸; Macchi, 2020 ▸; Macchi, 2022 ▸). There was little success in this respect until the Hansen–Coppens model (Hansen & Coppens, 1978 ▸) was put forward and rapidly adopted by the community (Gatti & Macchi, 2012 ▸). Thanks to their pseudo-atomic multipolar model, an unprecedented level of accuracy for experimental 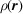 is now possible. The Hansen–Coppens model has also been adapted, with similar success, to the reconstruction of spin magnetic density from polarized neutron diffraction (PND) (Gillon & Becker, 2012 ▸; Brown *et al.*, 1979 ▸). Furthermore, combining both XRD and PND information turned out to be possible and allowed the reconstruction of the spin-resolved position-space electron distribution in ferromagnetic compounds (Deutsch *et al.*, 2014 ▸; Voufack *et al.*, 2019 ▸).

### 3.2. A momentum representation of electrons by Compton scattering

A. H. Compton’s 1923 experiment was another milestone in the construction of quantum mechanics (Compton, 1923 ▸). Just like X-ray diffraction (momentarily) settled the dispute between W. H. Bragg and C. G. Barkla (Stuewer, 1971 ▸), demonstrating the electromagnetic wave nature of X-rays, Compton’s results showed that X-rays could also behave as particles (Compton, 1925 ▸). The wavelength shift observed after X-rays had been scattered from a graphite sample, now called the Compton shift, was clear evidence that some kind of a photon–electron mechanical collision was involved. Nevertheless, we owe to J. DuMond the fact that, today, Compton scattering can be employed to better understand electron dynamics. Building upon the first elements of a theory proposed by de Broglie (de Broglie, 1926 ▸), and an innovative experimental setup, DuMond published a series of seminal papers interpreting the breadth of the shifted peak (hereafter denominated ‘Compton profile’) in terms of the electron momentum-space distribution (DuMond, 1928 ▸; DuMond, 1933 ▸). Amongst the reported experimental results was the early evidence that the electron gas is better described by Fermi’s statistics than the classical Boltzmann picture.

Compton scattering of X-ray photons is an inelastic incoherent process. As such, it is a radically different interaction mechanism from X-ray diffraction (Schulke, 2007 ▸). It has been shown that, in the limit of large energy and momentum transfer between the incoming photon and the electron, the signal is simply proportional to the so-called directional Compton profile 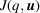 (DCP) defined by
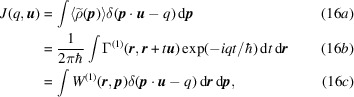
where 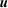 is the unit vector pointing along the scattering vector and 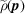 is still the one-electron probability density distribution in momentum space. It is under this assumption, known as the impulse approximation (Platzman & Tzoar, 1965 ▸), that a Compton scattering spectrum can be associated with 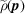 in such a straightforward manner (Cooper *et al.*, 2004 ▸). Expressions (16*a*[Disp-formula fd16]) and (16*c*[Disp-formula fd16]) clearly illustrate that a Compton spectrum measures the electrons’ marginal probability density distribution along the scattering vector. It is thus a projection of the momentum-space electron density onto a particular direction given by 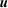. As such, expressions (16) are similar to the Radon transform encountered in many imaging techniques. Obtaining 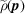 from a limited number of DCPs associated with different 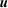 is still a challenging problem (Hansen, 1980 ▸; Dobrzyński & Holas, 1996 ▸; Gillet *et al.*, 1999 ▸; Kontrym-Sznajd & Samsel-Czekała, 2000 ▸).

R. Weiss (Weiss, 1969 ▸) and M. Cooper (Cooper *et al.*, 1965 ▸) pioneered the quantitative studies in momentum space. As early as 1969, they expressed that, as an incoherent process with large energy transfer, Compton scattering differs significantly from X-ray diffraction by two major aspects worth mentioning. The technique is only moderately affected by the crystal quality and only insofar as it changes the direction of chemical bonds from one site to another or significantly modifies the electron velocity distribution compared with the perfect crystal. Additionally, DCPs are barely perturbed by moderate temperature changes in the system (Matsuda *et al.*, 2020 ▸).

The downside of Compton measurements is that momentum-space distribution interpretations are not as direct as in position space because atomic contributions cannot be easily separated. Consequently, comparing Compton profiles with *ab initio* computations remains the most frequent practice. Furthermore, the general shape of directional Compton profiles is usually much less informative than Compton anisotropies, which are the differences between two directions in the Compton spectra or between each direction and the spectrum obtained from a reference powder sample. Compton anisotropies are widely accepted as the best way to remove less interesting core-electron contributions and most systematic errors. Two exceptions, where direct information is made accessible, are worth mentioning. With the development of high-resolution spectrometers on synchrotron sources, the shape of the Fermi surface in several metals became a natural outcome of Compton scattering measurements (Sakurai *et al.*, 1995 ▸; Hämäläinen *et al.*, 1996 ▸; Itou *et al.*, 1998 ▸) since, for ideal metals, the Compton profile takes the shape of an inverse parabola, touching the horizontal axis at the Fermi momentum in the chosen direction (Hämäläinen *et al.*, 2000 ▸). High-resolution Compton scattering has thereby played an important role in Fermiology (Bansil & Kaprzyk, 1997 ▸). Since electron dynamics observed by Compton scattering are moderately affected by crystal quality, atomic substitution effects can also be envisaged. The subtraction of DCPs between crystals of slightly different stoichiometry has thus paved the way for momentum-space orbital imaging. A notable example is the doped hole imaging in a high-*T*_c_ superconductor reported by Sakurai *et al.* (2011 ▸). Similar momentum information, with better core-valence discrimination, can be obtained from positron-annihilation spectroscopy, although the necessity to account for the positron wavefunction brings the data treatment to another level of complexity (Itoh *et al.*, 1982 ▸; Schulke, 2007 ▸). Finally, much like neutron diffraction, retaining only the unpaired electron’s contribution becomes possible if circularly polarized X-ray photons are used. Thereby, one also has access to the spin-magnetic density distribution of electrons in momentum space, 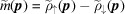. A review is given by Cooper *et al.* (2004 ▸), and a comparison between position and momentum representations for unpaired electrons can be found in Yan *et al.* (2017 ▸).

## 4. One-electron density matrices from experiments

Although a term invented by J. Karle (Massa, 2017 ▸), Quantum Crystallography (QCr) (Grabowsky *et al.*, 2017 ▸; Genoni *et al.*, 2018 ▸; Genoni & Macchi, 2020 ▸; Macchi, 2020 ▸; Macchi, 2022 ▸) was born with the pioneering works of pure-state 1-RDM reconstruction conducted by Clinton and coworkers (Clinton *et al.*, 1969*a* ▸; Clinton *et al.*, 1969*c* ▸). The same year, without explicitly seeking for a possible 1-RDM, Stewart (1969*a* ▸) also proposed a similar refinement of a charge–bond-order matrix from X-ray diffraction data.

It took almost forty years for QCr to become the umbrella term we use today. It now gathers research on electron charge or spin densities in position or momentum spaces, using X-ray, neutron or electron scattering techniques and relevant *abinitio* computational approaches. The term was rediscovered after Jayatilaka’s method (Jayatilaka, 1998 ▸), known as the X-ray restrained wavefunction (XRW), was published. It became even more broadly used when the combination of XRW with Hirshfeld atom refinement (Jayatilaka & Dittrich, 2008 ▸; Capelli *et al.*, 2014 ▸) became popular (Genoni & Macchi, 2020 ▸; Genoni, 2024 ▸).

An essential feature needs to be exposed at this stage. Experimental determination of a 1-RDM turns out to be significantly more challenging than position-space charge density. While any charge density model fitted on experimental data is physically acceptable as long as it accounts for all electrons in the system (*i.e.*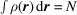), it was observed by Coleman (Coleman, 1963 ▸; Erdahl & Smith, 1987 ▸) that, if no particular precaution was taken, a model reduced density matrix is likely to yield unrealistic electronic energy values. It thus became a matter of utmost interest to determine the criteria for discriminating physically acceptable 1-RDMs. Coleman found the limited number of conditions under which a possible 1-RDM can be related to a pure-state wavefunction, or a statistical mixture of pure-state wavefunctions, of the *N*-electron assembly. These are called the *N*-representability conditions (Coleman, 1961 ▸; Coleman, 1963 ▸). In the 1-RDM case, there are still no practical ways of formulating the constraints for systems of realistic size (more than ten orbitals) if we restrict the electrons to be in a pure state (Altunbulak & Klyachko, 2008 ▸) For an ensemble of states, the *N*-representability constraints are more easily expressed as 
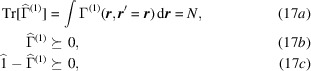
where 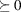 means that all the eigenvalues are non-negative. Then equation (17) amounts to retaining only the 1-RDMs that account for all electrons (17*a*[Disp-formula fd17]) but also with eigenvalues between 0 and 1 [(17*b*[Disp-formula fd17]) and (17*c*[Disp-formula fd17]), respectively]. Conditions (17) also ensure that the 1-Wigner obtained from such a 1-RDM is physically acceptable.

### 4.1. Pure states from X-ray diffraction

As previously mentioned, the crystallography community initiated the quest for an experimental electron density matrix determination. The main reason was that X-ray diffraction data were considered the best information source available for this purpose. However, X-ray structure factors give limited access to the 1-RDM, only through its (spin-traced) diagonal elements in position space. Namely, re-examining (15*b*[Disp-formula fd15]),
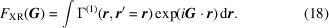
With such a strategy, no experimental information is exploited to determine the off-diagonal part of the matrix (*i.e.* when 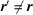). Moreover, as explained by Aleksandrov and coworkers (Aleksandrov *et al.*, 1989 ▸), there are an infinite number of 1-RDMs corresponding to a given density (Gilbert, 1975 ▸; Harriman, 1990 ▸; Harriman, 1992 ▸) and an unconstrained 1-Wigner may reveal to be pertinent only in the position portion of phase space.

A powerful strategy, inspired by McWeeny’s purification scheme (McWeeny, 1960 ▸), was therefore developed by Clinton *et al.* (Clinton *et al.*, 1969*b* ▸; Clinton & Massa, 1972 ▸). The procedure is generally to focus on the quest for a population matrix 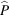, which is the discrete representation of the 1-RDM in a given basis set of functions 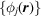 such that the 1-RDM can be written as
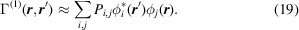
This can be seen as an approximate reformulation of (14) when the wavefunctions are expressed in terms of a limited set of orbitals.

The idempotent 1-RDM, also called the Fock–Dirac density matrix (FDRDM), must obey the Clinton equations, which impose asymptotic idempotency to the 1-RDM and the best fit against the chosen X-ray structure factors. The idempotency condition is defined as the mathematical projector property 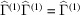. The *N*-representability conditions are automatically fulfilled since the eigenvalues of the resulting idempotent 1-RDM are necessarily 0 or 1. However, this very strict constraint forbids access to non-pure quantum states. Furthermore, it limits the model in its capacity to describe correlation effects, beyond those induced by Pauli’s principle (Fermi correlation), since it becomes equivalent to imposing a single-determinant *N*-electron wavefunction (Schmider *et al.*, 1990 ▸). Nevertheless, the Clinton equation approach was successfully tested not only for ‘simple’ systems (Massa *et al.*, 1985 ▸) but also molecular crystals (Howard *et al.*, 1994 ▸; Huang *et al.*, 1999 ▸). A very interesting alternative was put forward by Snyder & Stevens (1999 ▸), who conducted an idempotent-constrained density matrix refinement on experimental X-ray structure factors on a molecular compound without resorting to Clinton’s equations. Beyond their analysis of the resulting deformation density, the point worth noting is their comparison of the FDRDM-derived Hartree–Fock energy with the *abinitio* one. However, no explicit results for the kinetic component were given, leaving doubts about the detailed nature of the non-diagonal part. In a similar spirit of determining the FDRDM, Aleksandrov *et al.* first developed an alternative method for larger molecules and, more importantly, took a linear combination of atomic orbitals formalism extending the previous isolated-molecule model paradigm to other systems (Aleksandrov *et al.*, 1989 ▸). This paper is important because, to our best knowledge, it is the first work on the FDRDM with an explicit comparison of resulting directional Compton profiles with experimental data (for diamond and silicon). It is, therefore, a unique example of assessment of the pure-state 1-RDM model by a clear examination beyond its diagonal elements.

Jayatilaka’s X-ray restrained wavefunction (XRW) approach is an even more directed alternative to that presented by Clinton, Massa *et al.* Instead of using McWeeny’s purification scheme simultaneously with the XRSF fitting condition, this strategy developed from the original idea put forward by Henderson & Zimmerman (1976 ▸) and aims at minimizing the total energy of a trial *N*-electron wavefunction model in the usual variational manner (generally expressed as a single determinant) while enforcing the best possible reproduction of the XRSFs. The method converges towards an admissible *N*-electron wavefunction more efficiently, giving access to an idempotent 1-RDM. Thus, it can be seen as imposing a specific environment (using a new, data-conditioned, effective potential included in the Hartree–Fock or Kohn–Sham equations), represented by the information in the experimental XRSFs. Today, the method has gathered a significant community of users, mostly because of its versatility and, more specifically, its efficacy in predicting properties in important molecular compounds.

Given the importance of these methods for retrieving an experimental 1-RDM, one can only regret that no graphical representation of such an idempotent experimental 1-RDM has ever been published and could be compared with theoretical results at different levels of quantum chemistry computation. It can be assumed that because of the significant role played by the self-consistent field component, upon which X-ray restraint is a mere perturbation, the resulting pure-state 1-RDM should be very good quality.

Since one of our concerns in this article is to study the possibility of exploring the phase-space behaviour of electrons, it should be noted that the connections between the 1-RDM work described in this section with momentum-space properties are very sparse. To our knowledge, kinetic energy, which is proportional to the second moment of momentum-space density, has been briefly mentioned on only a few occasions. The first was by Massa *et al.* (1985 ▸), when they determined a 1-RDM for beryllium metal from fewer than 60 structure factors (Larsen & Hansen, 1984 ▸). These are the same data exploited in Jayatilaka’s 1998 ▸ seminal paper. As previously mentioned, more detailed momentum-space results were given in 1989 ▸ by Aleksandrov *et al.* (1989 ▸), after they obtained an idempotent 1-RDM for diamond and silicon. While the general behaviour of a unique directional Compton profile was evidence of a global qualitative agreement, the remaining discrepancies at low momentum indicate that the most delocalized chemical features were not fully described. Compton profile anisotropies would probably have been more informative in distinguishing the isotropic core-electron from the useful valence-electron contributions.

### 4.2. Pure states, statistical mixtures and combination of scattering methods

Since the only information used by the previous methods originates from X-ray diffraction structure factors, the difficult challenge is to determine a matrix while only its diagonal elements are (possibly) available. From an equivalent but different point of view, the lack of experimental input on the momentum distribution precludes reliable access to the 1-Wigner. To our knowledge, this is why no attempts to explore the quantum behaviour of crystal electrons in phase space from an experimental perspective have been reported yet. This lack of information also explains why the 1-RDM model needs to be heavily constrained at the price of imposing idempotency.

Schmider *et al.* (1990 ▸) observed that the momentum-space electron density distribution is directly connected to the density matrix’s off-diagonal elements. More precisely, directional Compton profiles as defined in equation (16) can easily be related to the 1-RDM since, for the scattering vector direction 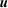, the DCP is given by (16*b*[Disp-formula fd16]). Equivalently, one could consider the auto-correlation function 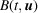, also named the reciprocal form factor, of which the inverse Fourier transform is the DCP 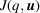:
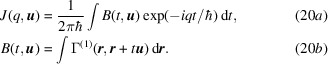
The reciprocal form factor has been particularly useful as an intermediate quantity for momentum-space density reconstruction from a set of DCPs (Hansen, 1980 ▸; Gillet *et al.*, 1999 ▸). 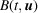 was first considered (Calais, 1981 ▸) for chemical bond characterization before Bader’s topological method was broadly adopted.

A closer examination of (16*b*[Disp-formula fd16]) and (18) can help to understand how information from a large set of DCPs is a precious supplement to X-ray diffraction for 1-RDM experimental determination.

Reports of direct wavefunction, 1-RDM or 1-Wigner extraction from solely momentum-space-oriented experiments are sparse. A very early attempt was published by Pecora (1986 ▸), who modified Clinton’s method and explained how it could be employed to exploit positron-annihilation-derived momentum density data better. They first demonstrated that such momentum-space information could retrieve minute chemical details, which usually go undetected by other methods. This triggered a series of works in which momentum space and Compton data were precious experimental information sources. Simple wavefunction models were refined from a large set of DCPs for LiH (Gillet *et al.*, 1995 ▸) or MgO (Gillet *et al.*, 2001 ▸). Perhaps even more relevant to our subject are the 1-RDM reconstructions for atomic systems reported by Schmider (Schmider & Smith, 1993 ▸). The latter group initiated a series of studies where several strategies were considered to obtain a non-necessarily idempotent 1-RDM from a combination of X-ray scattering data, mostly on atomic systems, to push the description further than the single-determinant model (Schmider *et al.*, 1990 ▸). While no graphical illustration of the total 1-RDM thus obtained was offered, the final results strongly advocated combining Compton and diffraction data to reach the most accurate picture of electron behaviour in these atomic systems. The question of tackling the delicate problem of rendering a thorough phase-space picture of electrons in a real molecular (or crystalline) system was still to be solved.

## 5. Combining data for a phase-space representation of chemical bonds

As explained in the previous sections, the quest for an experimental 1-RDM is not a new topic and has accompanied the development of QCr since the late 1960s. Nevertheless, for the following five decades, the difficulties or successes in obtaining satisfying results were seldom judged by observing the graphical representation of the 1-RDM itself. Noticeable exceptions are those given in Schmider *et al.* (1992 ▸) and Schmider *et al.* (1993 ▸) but did not attract the attention they deserved. Even in the QCr community, few knew what a density matrix looked like. Furthermore, since the phase space was globally ignored, except for the examples published by Springborg (Dahl & Springborg, 1982 ▸; Springborg, 1983*a* ▸) in the atomic case and Springborg (1983*b* ▸) in the molecular LiH case, the 1-Wigner representations are virtually absent from the QCr literature. In the remaining part of this article, emphasis will be placed on the discrepancies, and sometimes agreements, that the reconstructed 1-RDM and 1-Wigner can have with *abinitio* results.

Starting with a paper reinstating the importance of combining elastic and inelastic scattering data (Gillet, 2007 ▸), our group has been active during the last two decades in exploring different methods for recovering the 1-RDM in molecules and crystals from the above-mentioned experiments. Two different paths have been considered. What is the best strategy for combining data, and what is the most pertinent 1-RDM model? In line with most previously reported works, ensemble *N*-representability is the first necessary condition that any viable 1-RDM model must fulfil. From the start, it was decided not to consider the idempotency option, which had been extensively explored since McWeeny and Clinton’s works and would be an obstacle to the thorough account of electron correlation effects. As a result, allowing the 1-RDM eigenvalues to take intermediate values between 0 and 1 can thus be made possible in at least two ways. The easiest is probably to compute, by an *abinitio* method for a given basis set, the 1-RDM and its natural orbitals (*i.e.* its eigenfunctions), then refine the occupation numbers (*i.e.* its eigenvalues) to find the best fit against experimental data. A more challenging alternative is still choosing a basis set and then refining the full population matrix subject to *N*-representability constraints. For simplicity’s sake, we now assume a set of orthogonal basis functions. This basis set is constructed from an extensive set of atom-centred Cartesian Gaussian functions. Thus we will denote the population matrix in the orthogonal basis as 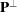. Only spin-paired electron systems are considered here, but an extension to spin-resolved experiments is straightforward. The global formalism can be summarized as the following optimization procedure, starting from expression (19) then finding the elements 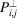 of matrix 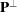 that minimize the statistical agreement function (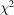) between experiment and model prediction:
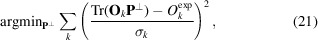
where *k* sums over all experimental data and 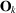 are the observable operator matrices corresponding to experimental data values 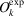 with standard deviation 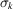. Assuming only spin-paired electrons, optimization (21) is subject to
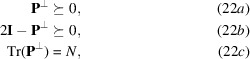
where (22*a*[Disp-formula fd22]) and (22*b*[Disp-formula fd22]) constrain the eigenvalues to be found in the 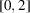 interval, while (22*c*[Disp-formula fd22]) fixes the sum of occupation numbers to match the total number of electrons in the system.

In keeping with the preceding sections, only XRSFs and DCP experimental data have been used throughout these works. Therefore, one can represent the matrix elements of 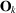 as either
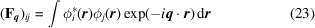
or
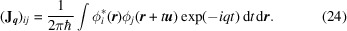
To account for possible thermal agitation, each XRSF matrix element can be modified by the adjunction of a Debye–Waller (DW) factor of the form 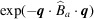, where *a* refers to the nucleus centre to which both basis functions are attached. When the basis functions do not share the same nucleus centre, different schemes can be used to average the Debye–Waller factors of two nuclei (Stewart, 1969 ▸; Coppens *et al.*, 1971 ▸; Tanaka, 1988 ▸). For example, in the Coppens model the average is taken as 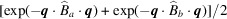. In the last reported work (Yu & Gillet, 2024 ▸), only single-centre DW terms are used to avoid unfair similarity with the computational method used to generate the reference data.

The model that determines all elements of the population matrix is the most flexible. It is also the most difficult to constrain *N*-representability beyond idempotency [as in conditions (22)]. Fortunately, semi-definite programming methods and packages have become available, and preliminary tests on toy systems have demonstrated their potential (Mazziotti, 2007 ▸; De Bruyne & Gillet, 2020 ▸). The compatibility of a convex optimization approach with the highly non-linear determination of the thermal agitation contribution was further tested with success in Launay & Gillet (2021 ▸). Therefore, after having experience of the occupation number refinement in previous works (Gueddida *et al.*, 2018*a* ▸; Gueddida *et al.*, 2018*b* ▸), and confirming the difficulty in providing the necessary flexibility, it was decided to give pre-eminence to the 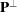 refinement.

**Figure 1:**
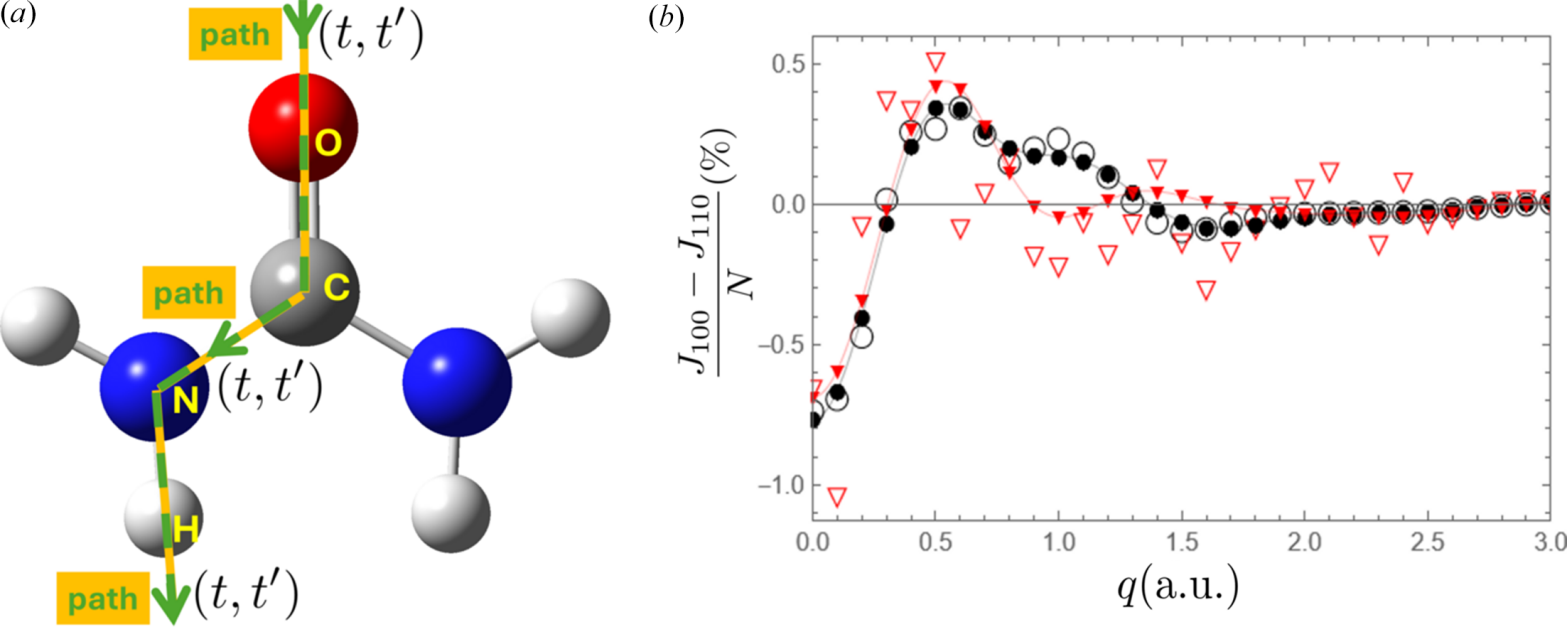
(*a*) The dashed line gives the path through the urea molecule. It is the one-dimensional position space used to represent the 1-RDM or the 1-Wigner, with *t* and 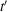 the curvilinear coordinates. (*b*) Compton anisotropies (in % of an electron) comparing the DCPs along the 100 and the 110 directions in the urea crystal. Empty circles: 3D *abinitio* calculations. Empty triangles: artificial data [same as before but corrupted with noise]. Connected black dots: model refined from ideal data (no noise, DCPs and XRSFs at 0 K). Connected filled red triangles: model refined from artificial data (with noise, DCPs at 0 K and XRSFs at 52 K).

The latest compound under scrutiny is urea, [CO(NH_2_)_2_], shown in Fig. 1 ▸(*a*). Details of this work have been reported by Yu & Gillet (2024 ▸). Despite the lack of a centre of symmetry, the crystal has been the subject of several high-resolution X-ray diffraction measurements (Zavodnik *et al.*, 1999 ▸; Birkedal *et al.*, 2004 ▸) and some of the very rare Compton measurements on molecular crystals (Shukla *et al.*, 2001 ▸). The latter exhibit weak Compton anisotropy, which is expected to become another difficulty, as mentioned in Section 3.2.

First, both periodic and molecular *abinitio* calculations for urea are carried out with density functional theory on a B3LYP/POB-DZVP (Peintinger *et al.*, 2013 ▸) level of theory using, respectively, the *Crystal* code (Dovesi *et al.*, 2014 ▸) and *Gaussian 16* (Frisch *et al.*, 2016 ▸). From the periodic calculation, and its resulting theoretical 1-RDM, 3627 XRSFs at 0 K and 52 K, and DCPs along eight directions are computed to serve as reference data for the refinement. The 52 K Bragg and Compton data are then corrupted with statistical noise. As a common practice for position- and momentum-space density reconstructions, the core-electron contribution was pre-calculated by a standard *abinitio* procedure and kept frozen throughout the 1-RDM modelling process. All symmetry invariances of the urea molecule were also imposed to construct the single-molecule 1-RDM model. Translation and rotational operations were applied to fully recover the crystal-phase properties. However, it must be made clear that the model only accounts for a single molecule in its environment and does not involve any Bloch function or intermolecular contribution.

**Figure 2:**
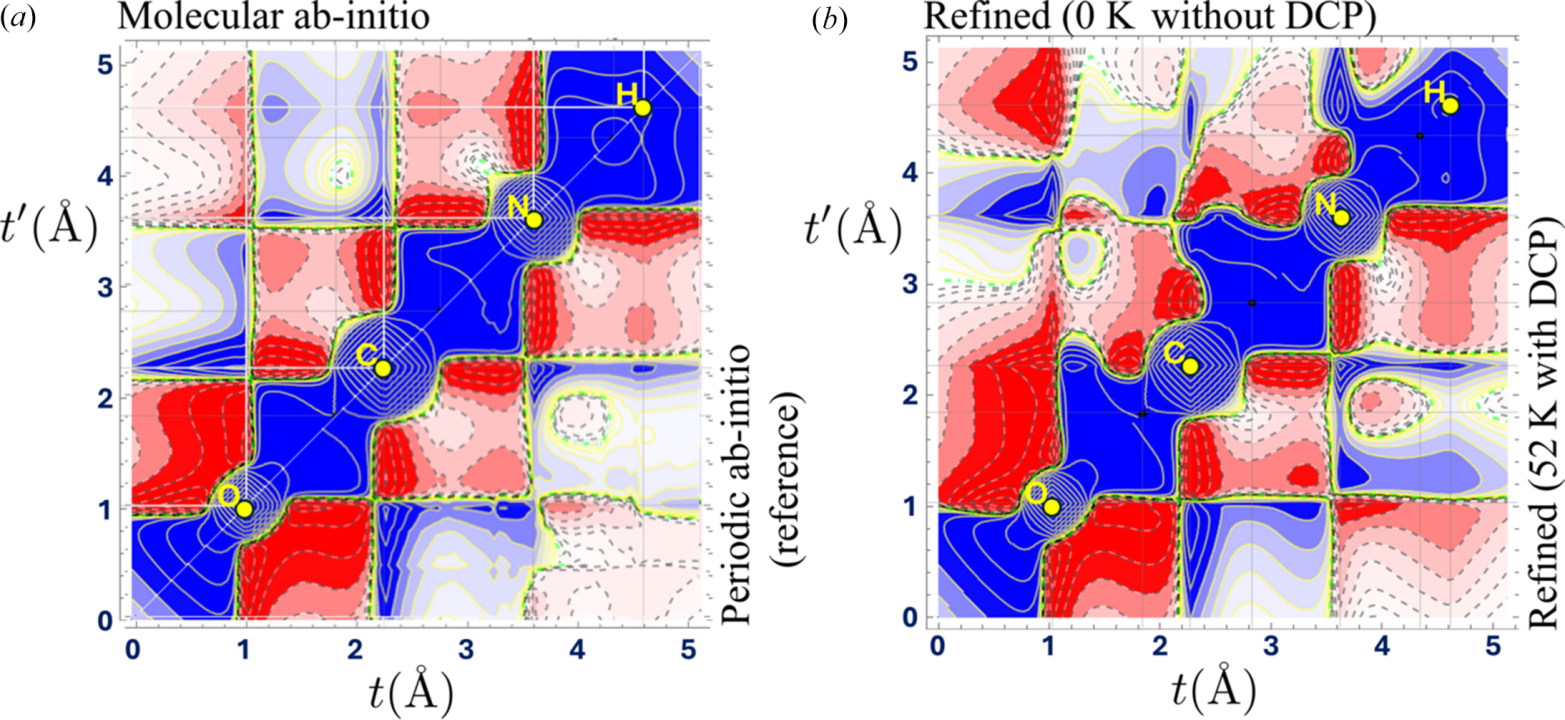
(*a*) Theoretical 1-RDM computed along the path given in Fig. 1 ▸. The upper portion of the matrix gives the isolated-molecule case. The lower portion corresponds to the periodic *abinitio* calculation used for generating the artificial data. (*b*) The upper portion gives the 1-RDM model refined using only 0 K XRSFs. The lower portion shows the same model but refined using a combination of noisy DCPs and XRSFs (at 52 K). Contours (positive solid blue) are every ±0.01 × 2^*n*^ e Å^−3^, with 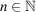.

To assess the quality of the refinement from the 1-RDM perspective, there is no alternative but to use artificial data computed from a known *abinitio* 1-RDM. This procedure is particularly useful for evaluating the ability of the model to gather information from both sets of data, XRSFs and DCPs. Fig. 2 ▸(*a*) displays both the periodic and molecular *abinitio* 1-RDM (Dovesi *et al.*, 2014 ▸). Fig. 2 ▸(*b*) shows the model 1-RDM with a 6-31G(p) basis set refined from a set of 3627 XRSFs when 8 DCPs are included (upper left triangle) or left out (lower right triangle). More specifically, the XRSF data are at 0 K and noise-free when the DCPs are left out (lower right) to highlight the impact of Compton data alone. When all data are used, both the XRSFs and DCPs are corrupted with noise and the XRSFs are simulated at 52 K. More detailed analysis regarding noise and temperature effects can be found in Yu & Gillet (2024 ▸).

At this stage, knowing how to interpret such pictures may be useful. Firstly, the density matrix for a 3D system is a function from 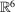 to 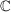 (or to 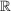 if only real basis functions are chosen to describe stationary states). Consequently, the graphical representation on a 2D page necessitates some drastic reduction, either by projection or a cut. This is somewhat arbitrary and depends on what features are found to be the most relevant. Here, we opted for a cut and followed a path constructed from a list of segments connecting the successive nuclei positions O–C–N–H [Fig. 1 ▸(*a*)]. With this choice, one can only access σ-electrons but pass through each bond critical point (Bader, 1990 ▸).

The representation thus gives the values of a continuous matrix 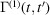 where *t* and 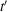 are curvilinear coordinates along the chosen path. When 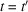 the value corresponds to that taken by the electron density in the molecule at this specific position on the path. Note that this matrix representation is flipped upside-down compared with the usual matrix notation. The contours on the diagonal (thus from bottom left to top right) are positive, just as a probability density should be. A detailed description of specific regions is proposed by Sandoval-Lira *et al.* (2016 ▸). We can mention here that off-diagonal parts describe the relationship between distant regions and how it affects the behaviour of the mean electron as a representative of the system’s global *N*-electron wavefunction(s). Therefore, it is an accurate describer of long-range interactions at work in the chemical bonding mechanism.

To better test the stability of the refinement process, nuclei agitation at moderate temperature can be included in the artificial data (Erba *et al.*, 2013 ▸) together with random noise [see Yu & Gillet (2024 ▸) for details of the noise generation]. In the urea case, it was found that Compton anisotropies tend to be buried under any realistic noise level [Fig. 1 ▸(*b*)]. Consequently, the refinement becomes more challenging. This is seen in Fig. 2 ▸(*b*) where second-neighbour interactions, such as between O and N, are (moderately) deteriorated. But changes can also be observed in regions corresponding to the interaction between both sides of the C or N nuclei. Additionally, the off-diagonal part is closely connected to the momentum density (16*a*[Disp-formula fd16]) and, as such, determines *T*, the kinetic energy of the system. It is thus instructive to estimate the resulting virial ratio 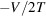 after the refinements shown in Fig. 2 ▸. If the two-electron potential energy *V* is computed from the 2-RDM expression ansatz 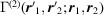 = 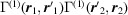 − 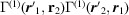, it is found that the estimated virial ratio from a refinement using both data sets only deviates from unity by 0.4% (against 6.6% when the 8 DCPs are omitted).

**Figure 3:**
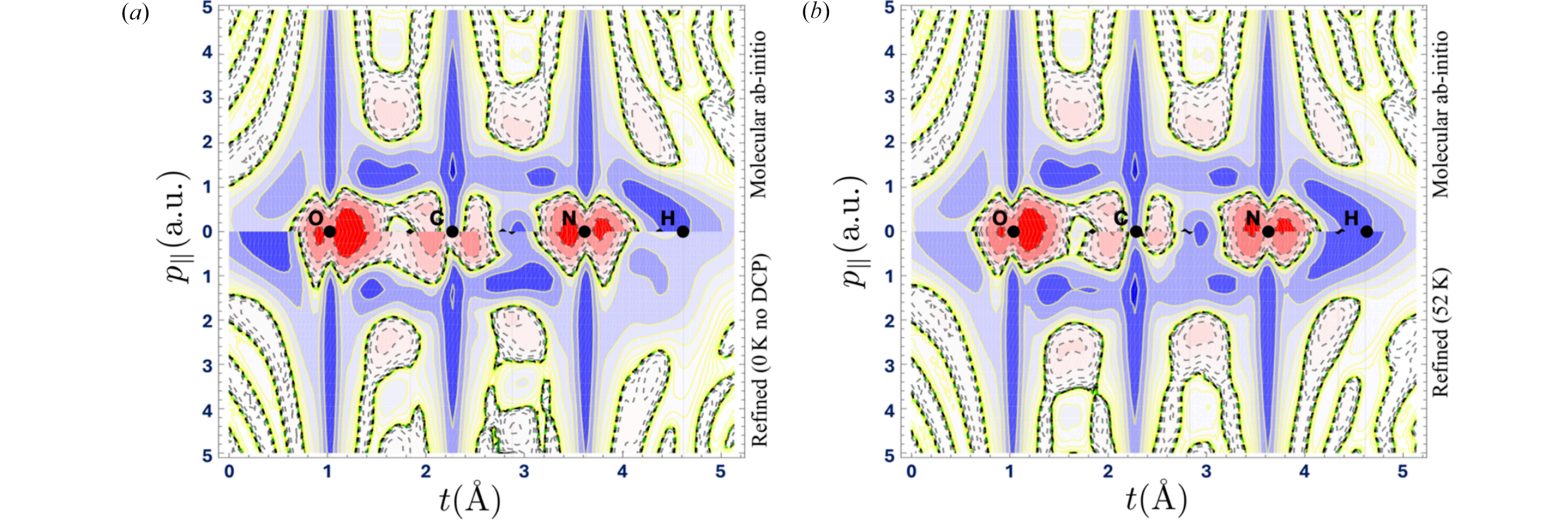
The 1-Wigner along the path given in Fig. 1 ▸(*a*). The vertical axis indicates the values of the momentum 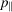 (in a.u.) tangent to the path at the current curvilinear position *t*. In both panels, the upper half is the *abinitio* 1-Wigner for the isolated molecule. The lower half in (*a*) is the refined 1-Wigner model from XRSFs at 0 K and without DCP data [same refinement as Fig. 2 ▸(*b*) upper triangle]. The lower half of (*b*) is the same refined model but with a combination of artificial data [same refinement as Fig. 2 ▸(*b*) lower triangle]. Contours (positive solid blue) are every ±2^*n*^ × 10^−7^ e (a.u. Å)^−3^, with 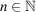.

Following expression (4), the 1-Wigner can be deduced from the refined 1-RDM. The results are reported in Fig. 3 ▸. The expected *ab initio* 1-Wigner along the chosen path is displayed in the upper portion. Not surprisingly, the long vertical blue stripes confirm that the electrons that are the closest to the nuclei (O–C–N) are also those with the possible highest momentum values along the path. This can be related to the Heisenberg position–momentum inequalities. But another significant manifestation of quantum behaviour is the negative (red-dashed) more diffuse features. Their existence is a consequence of interference between atomic contributions and disqualifies the 1-Wigner as a position–momentum joint probability distribution while labelling its quantum origin. The impact of Compton profiles is evident when comparing the 1-Wigner function reconstructed using all available artificial data with that obtained using only artificial structure factors. In the high-momentum regime (*p*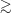 2 a.u.), the lower part of Fig. 3 ▸(*b*) exhibits better overall similarity with the molecular DFT calculations shown in the upper part, even if the data are affected by noise and thermal smearing. This contrasts with the reconstruction using only structure factors and displayed in the lower part of Fig. 3 ▸(*a*). In the low-momentum regime (*p*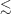 2 a.u.), the absence of Compton profiles leads to the omission of specific features in the reconstructed 1-Wigner function. For instance, the sign change between the carbon and oxygen atoms, absent in the lower part of Fig. 3 ▸(*a*), is successfully recovered in Fig. 3 ▸(*b*). Another striking difference occurs around the hydrogen atom. The reconstruction without Compton profiles fails to capture features behind the final H atom, as shown in Fig. 3 ▸(*a*). This is due to the limited information that can be extracted from the structure factors for this region of space. In contrast, Fig. 3 ▸(*b*) demonstrates qualitative agreement around the hydrogen atom when Compton profiles and structure factors are jointly employed. These figures are thus a clear demonstration that a combination of data related to the projections of the 1-Wigner in position (via XRSFs) and momentum (via DCPs) spaces opens up the gates of phase space to a quantum representation of electrons in crystals.

## 6. Conclusion

More than a century after their key role in the emergence of quantum physics, X-ray diffraction and Compton scattering are still powerful techniques for providing access to the most profound and fundamental electron behaviour in crystalline systems. Such a combination of experimental methods, which has become one of the pillars of quantum crystallography,remains in the early phases of development. The success of the model and strategy described in this paper supports the idea that phase space, once pictured as the sole territory of classical physics, could shortly become accessible for real systems as complex as electrons in molecules and crystals.
